# The role of TRIM24 during prostate cancer progression

**DOI:** 10.1186/1756-8935-6-S1-P24

**Published:** 2013-03-18

**Authors:** Anna C Groner, Myles Brown

**Affiliations:** 1 Division of Molecular and Cellular Oncology, Department of Medical Oncology, Dana-Farber Cancer Institute and Department of Medicine, Brigham and Women’s Hospital and Harvard Medical School, Boston MA 02115, USA

## Introduction

The steroid hormone androgen mediates a wide range of developmental, physiological and malignant responses by acting as a ligand for the androgen receptor (AR). AR in turn functions as a nuclear receptor transcription factor and executes specific gene expression programs in an androgen-dependent manner. Since the growth of prostate cancer cells initially depends on androgen, cancer therapy uses hormone-deprivation approaches to reduce the levels of serum androgen. While prostate cancer (PC) initially responds to androgen-ablation, most tumors progress to a castration resistant (CR) state insensitive to treatment. Both the androgen-dependent and the CRPC state depend on AR, which is probably activated by alternative molecular pathways in CRPC in response to low androgen levels.

## Methods and results

Here, we assess the role of the transcriptional co-regulator TRIM24/TIF1α in mediating AR-dependent gene expression and growth. We find that TRIM24 expression is increased in different states of PC, particularly in CRPC. When we induce the drug-regulated knock-down of TRIM24, we find that cell growth in both our androgen-dependent (LNCaP) and in our CRPC model (LNCaP-abl) is affected. Therefore, we hypothesize that TRIM24 is involved in mediating both ligand-dependent and ligand-independent activation of AR. This is further consistent with our finding that TRIM24 and AR can physically interact in both conditions. We are currently identifying TRIM24-dependent gene expression programs by combining genome-wide binding analyses with microarray studies.

## Discussion

This will help us shed light on how the transcriptional co-regulator TRIM24 may reprogram AR activity in its progression from an androgen-dependent to a castration-resistant state. Current results from this study will be presented at the meeting.

